# Low-Complexity Multiple Transform Selection Combining Multi-Type Tree Partition Algorithm for Versatile Video Coding

**DOI:** 10.3390/s22155523

**Published:** 2022-07-25

**Authors:** Liqiang He, Shuhua Xiong, Ruolan Yang, Xiaohai He, Honggang Chen

**Affiliations:** College of Electronics and Information Engineering, Sichuan University, No. 24 South Section 1, Yihuan Road, Chengdu 610065, China; heliqiang@stu.scu.edu.cn (L.H.); xiongsh@scu.edu.cn (S.X.); yangruolan@stu.scu.edu.cn (R.Y.); hxh@scu.edu.cn (X.H.)

**Keywords:** versatile video coding, multiple transform selection, fast intra-coding, CU partition, real-time sensor networks

## Abstract

Despite the fact that Versatile Video Coding (VVC) achieves a superior coding performance to High-Efficiency Video Coding (HEVC), it takes a lot of time to encode video sequences due to the high computational complexity of the tools. Among these tools, Multiple Transform Selection (MTS) require the best of several transforms to be obtained using the Rate-Distortion Optimization (RDO) process, which increases the time spent video encoding, meaning that VVC is not suited to real-time sensor application networks. In this paper, a low-complexity multiple transform selection, combined with the multi-type tree partition algorithm, is proposed to address the above issue. First, to skip the MTS process, we introduce a method to estimate the Rate-Distortion (RD) cost of the last Coding Unit (CU) based on the relationship between the RD costs of transform candidates and the correlation between Sub-Coding Units’ (sub-CUs’) information entropy under binary splitting. When the sum of the RD costs of sub-CUs is greater than or equal to their parent CU, the RD checking of MTS will be skipped. Second, we make full use of the coding information of neighboring CUs to terminate MTS early. The experimental results show that, compared with the VVC, the proposed method achieves a 26.40% reduction in time, with a 0.13% increase in Bjøontegaard Delta Bitrate (BDBR).

## 1. Introduction

At present, real-time sensor networks (e.g., Visual Sensor Networks (VSNs) and Vehicular Ad-hoc Networks (VANETs)) are rapidly evolving, with advances in imaging and micro-electronic technologies. These networks acquire multimedia data such as images and video sequences, integrating low-power and low-cost vision sensors. As a key application in sensor networks, video compression and transmission technologies are widely used in the field of broadcasting and communications. Furthermore, with the widespread use of 5th Generation (5G) mobile networks [1,2] and the rapid development of the Internet of Things (IoT) [3,4,5,6], technologies for the coding and transmission of multimedia information have become a popular research direction. Video sequences can be better integrated with real-time sensor networks by improving the performance of multimedia information compression. Hence, it is essential to investigate an efficient and fast video coding standard for the application of encoded videos in real-time networks.

With the development of high-resolution video applications, High Dynamic Ranges (HDRs), and High Frame Rates (HFRs), the urgent demand for a new generation of video-coding technologies, beyond the High-Efficiency Video Coding (HEVC) standard [7], has increased. The Joint Video Experts Team (JVET) has formulated the latest standard, called Versatile Video Coding (VVC) [8], to address this issue. The VVC relies on a series of high-computation coding tools to achieve a better coding performance than HEVC [9,10,11,12,13,14,15]. For intra-prediction, the Position-Dependent Intra-Prediction Combination (PDPC) [16,17] and Cross-Component Linear Model (CCLM) [18,19] are utilized to optimize prediction accuracy. Moreover, Sub-Block Transform (SBT) [20] and Low-Frequency Non-Separable Transform (LFNST) [21,22] are employed to further eliminate frequency redundancy. However, this high complexity limits the use of VVC in real-time multimedia applications.

Transform is one of the most important modules in the video-coding model, since the predicted residual blocks need to be transformed in all frames for subsequent quantization and entropy coding processes. On the basis of obtaining different orientation angles, the Steerable Discrete Cosine Transform (SDCT) [23] is proposed in HEVC to exploit the directional Discrete Cosine Transform (DCT). To further enhance the coding efficiency, the Multiple Transform Selection (MTS) [24] was proposed in the VVC, allowing for the encoder to select a pair of horizontal and vertical transforms from a predefined set. These sets include kernels from three trigonometrical transforms: DCT-II, Discrete Sine Transform Type VII (DST-VII) and DCT-VIII. The MTS candidate indexes and corresponding transform matrices are shown in Table 1. When the MTS in the Sequence Parameter Set (SPS) is enabled, RD checking will be performed on combinations of DST-VII and DCT-VIII in horizontal and vertical directions after applying DCT-II in both directions. With minimal Rate-Distortion (RD) costs, the VVC can determine the optimal transform during the Coding Unit (CU) partition and mode decision stages. Compared with HEVC, the computational complexity of the above process is increased as the RD cost of several transforms needs to be evaluated. Besides, many advanced coding tools have been adopted in VVC, such as the Quad-Tree plus Multi-Type Tree (QTMT) partition structure [25], the affine motion compensation prediction [26,27] and the 67 intra-directional prediction modes. These advanced tools make the coding process in VVC quite flexible while increasing the computational complexity. Although VVC achieves a better coding performance than HEVC, its complex computation greatly increases the coding time, which makes it difficult to use in real-time sensor application networks. Hence, it is necessary to simplify the coding process in VVC to make it suitable for real-time applications.

In this paper, we propose a low-complexity multiple transform combined with a multi-type tree partition algorithm to accelerate the VVC coding process. It is worth mentioning that the proposed algorithm could be combined with the fast CU partition method and other methods to achieve more significant computational reductions during the coding process.

The main contributions of this work are summarized as follows:

1 Different from previous studies that reduced the computation by terminating CU partition early, we propose a method to reduce computation complexity by investigating the MTS process, to make it more suitable for real-time applications than VVC.
2 An MTS skipping method is introduced by exploring the relationship between the RD cost of transforms and the correlation between Sub-Coding Units (sub-CUs) information entropy. The RD checking of MTS can be skipped by comparing the sum of the RD costs of the sub-CUs with the RD cost of their parent CU.
3 Based on the coding information of neighboring CUs, the MTS early-termination method is proposed to reorder the candidates in MTS for subsequent RD checking.

## 2. Related Work

Considering the low computational complexity of encoders can accelerate the coding and transmission of videos and achieve low-latency video streaming. Hence, a video encoder with high coding efficiency and low complexity is a core requirement for real-time sensor networks with limited transmission bandwidth and computational power.

Most previous works focused on the early termination of the CU partition to speed up the coding process. Based on the edge information extracted by the canny operator, Tang et al. [28] proposed a method for CU partition in intra- and inter-coding. Lin et al. [29] introduced a spatial feature method to accelerate the binary tree partition of CU. In [30], the depth information of adjacent CUs was used to determine the depth of the current CU partition. The position of reference pixels was utilized in [31] to minimize the coding complexity of the Intra Subpartition (ISP) tool. In [32], a fast intra method was proposed to reduce coding complexity by removing non-promising modes. Zhang et al. [33] proposed an entropy-based method to accelerate the CU partition. In [34], a fast block-partitioning method was proposed to skip the CU splitting and Rate-Distortion Optimization (RDO) process by using a Light Gradient Boosting Machine (LGBM). To extract and utilize features more efficiently, some methods of accelerating CU partition are proposed, based on the Convolutional Neural Network (CNN). For JVET intra-coding, Jin et al. [35] proposed a CNN-based fast-partition method. Similar studies have been conducted in [36,37]. By jointly using multi-domain information, Pan et al. [37] introduced a fast inter-coding method to terminate the CU partition process early. A Hierarchy Grid Fully Convolutional Network (HG-FCN) framework was proposed in [38] to effectively predict the quad-tree with a nested multi-type tree (QTMT). There are also several studies that focus on the fast algorithm of other coding tools. In [39], an entropy-based method was proposed to replace the standard rate estimation. In [40], the approximation of DCT-VII was modelled to reduce the computation. By combining the histogram of oriented gradient features and the depth information, Wang et al. [41] proposed a sample adaptive offset acceleration method to reduce the computational complexity in VSNs. Jiang et al. [42] used a Bayesian classifier for the inter-prediction unit decision. However, the studies on fast algorithms for MTS are rare. There is still much room for improvement to speed up the coding process.

This paper focuses on accelerating the MTS process in VVC to reduce coding time and meet the requirements of real-time applications. It is worth mentioning that the proposed method also can be combined with fast CU partitioning algorithms to further reduce coding complexity.

## 3. Materials and Methods

To accelerate the coding process in VVC, a low-complexity multiple-transform selection combined with a multi-type tree partition algorithm is proposed in this paper. First, based on the correlation between sub-CUs information entropy and the relationship between the RD cost of transforms, the RD cost of the last child CU can be estimated to reduce the computational complexity. Furthermore, if the sum of children CUs’ RD costs of the split pattern is greater than or equal to the RD cost of the parent CU, the RD checking of MTS will be skipped early. Second, based on the coding information of neighboring CUs, the MTS candidate checking is adaptively sequenced to make the RDO process more efficient, so that the RD cost-checking of MTS for selected intra-modes can be terminated earlier. The details are described as follows.

### 3.1. MTS Early Skipping Method

Based on the RD calculation for no splitting, horizontal binary splitting, vertical binary splitting, vertical binary splitting, horizontal ternary splitting, vertical ternary splitting and quad-tree splitting, the CU in VVC is successively partitioned. In the recursive RDO search process of CU partition, whether to split the current CU is determined by the RD cost of the current CU and its sub-CUs, as given by Formula (1):

(1) $$split\_flag=\left\{\begin{array}{c} 0,\;RD_{p}\leq \sum_{i=1}^{N}RD_{i},\\ 1,\;else, \end{array}\right.$$

where RD_p and RD_i represent the RD cost of the current CU and the i-th sub-CUs, respectively. *N* is the total number of the children CUs. split_flag indicates whether the current CU is split. When the sum of the children CUs’ RD costs of the split pattern is greater than or equal to the RD cost of the current CU, split_flag is set to 0, and the current CU will not be split. Otherwise, split_flag is set to 1, which means the current CU will be split.

The minimum RD cost of the last CU in Formula (1) is obtained by comparing the RD cost of primary transform and MTS. The Formula (1) can be written as:

(2) $$split\_flag=\left\{\begin{array}{c} 0,\;RD_{p}\leq \sum_{i=1}^{N-1}RD_{i}+min\left(RD\_pri,RD\_mts\right),\\ 1,\;else, \end{array}\right.$$

where RD_pri is the RD cost of primary transform for the last child CU. RD_mst represents the RD cost of MTS for the last child CU.

Therefore, the above process can be accelerated by estimating the RD cost of the last child CU. Moreover, the RD checking of the MTS will be skipped in advance under the condition that the sum of the RD costs of the sub-CUs of the split pattern are greater than or equal to the RD cost of their parent CU. To more accurately estimate the RD cost of the last child CU, we counted the probability of using the same optimal transform for two adjacent sub-CUs under binary splitting in video sequences of different resolutions. Table 2 shows the probability of using the same optimal transform in two adjacent sub-CUs under binary splitting for all frames in a portion of the test video sequences. The quantization parameter (QP) was set at 22, 27, 32, and 37. We can observe that the optimal transform of two sub-CUs is the same for most binary-splitting cases in video sequences of different resolutions. Furthermore, the two sub-CUs under binary splitting also have the same size. Hence, using the previous RD cost as the estimate of the last child CU under binary splitting is reasonable for sub-CUs with a strong correlation.

Considering the information entropy of CUs can help to effectively reflect their amount of content. Therefore, we used the information entropy of the two sub-CUs under binary splitting to measure their similarity. Specifically, in the proposed MTS early skipping method, we first calculated the information entropy *H* of i-th sub-CUs as follows:

(3) $$H_{i}=-\sum_{m=1}^{n}P\left(m\right)\times \log_{2}P\left(m\right),$$

where P(m) represents the probability of factor *m* in the i-th sub-CUs. *n* is the total number of the factors in the i-th sub-CUs.

Then, the similarity of the two adjacent sub-CUs was measured by the ratio of information entropy as follows:

(4) $$S=\frac{H_{2}}{H_{1}},$$

where $H_{1}$ and $H_{2}$ denote the information entropy of the previous and the last sub-CUs under the binary tree partition, respectively. If *S* is closer to 1, this means that the two sub-CUs are more similar.

**Figure 1:**
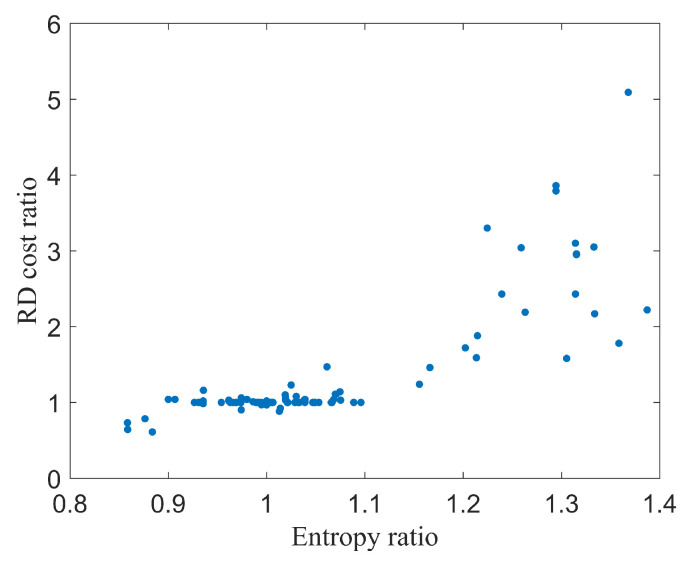
The relationship between RD cost ratio and entropy ratio of adjacent sub-CUs under binary splitting.

To analyze the relationship between the similarity and information entropy ratio of two sub-CUs, we counted the information entropy ratio and RD cost ratio of two sub-CUs under binary splitting. Figure 1 exhibits an approximate correlation between the information entropy ratio and the RD cost of two adjacent sub-CUs under binary splitting in a encoded frame. The QP was set at 27. This illustrates that, when the information entropy ratio *S* is in the range of 0.9 to 1.1, the two adjacent sub-CUs have a strong similarity and their RD costs are very close. Furthermore, there is a positive relationship between the RD cost and information entropy of adjacent sub-CUs. Thus, for two adjacent sub-CUs with high similarity, the RD cost of the last child CU can be estimated by the product of the previous RD cost and the information entropy ratio of the adjacent sub-CUs. The Formula (2) can be derived as:

(5) $$split\_flag=\left\{\begin{array}{c} 0,\;RD_{p}\leq \sum_{i=1}^{N-2}RD_{i}+\left(1+\frac{H_{2}}{H_{1}}\right)\times RD_{N-1},\\ 1,\;else, \end{array}\right.$$

As the CU content with quad-tree splitting in VVC is usually diverse and complex, the estimation of the final RD cost may not be accurate enough, leading to degradations in the coding performance. Hence, the proposed algorithm does not modify the MTS process in the case of quad-tree splitting. In [43], Fu et al. demonstrate that the RD cost of primary transform is approximately equal to the values of MTS in most cases. When the CU is split by ternary tree partition or the sub-CUs under binary splitting are dissimilar, only the primary transform is used to calculate the RD cost of the last child CU. The Formula (2) can be written as follows:

(6) $$split\_flag=\left\{\begin{array}{c} 0,\;RD_{p}\leq \sum_{i=1}^{N-1}RD_{i}+RD\_pri,\\ 1,\;else, \end{array}\right.$$

According to Formulas (5) and (6), when the split_flag is 0, the current CU is no longer split, so the RD checking of MTS can be skipped in the intra-coding process. The isSkipMTS is used to determine whether to skip the MTS. The details of the proposed MTS early skipping method are shown in Algorithm 1.
**Algorithm 1 **The proposed MTS early skipping method**Input: **$RD_{i}\left(1\leq i\leq N-1\right)$, $RD_{p}$, $H_{1}$, $H_{2}$ **Output: **isSkipMTS1:intialize isSkipMTS to false2:**if** partition mode is binary splitting and $0.9\leq \frac{H_{2}}{H_{1}}\leq 1.1$ **then**3:    **if** $RD_{p}\leq \sum_{i=1}^{N-2}RD_{i}+\left(1+\frac{H_{2}}{H_{1}}\right)\times RD_{N-1}$ **then**4:        isSkipMTS=true5:    **end if**6:**else if** partition mode is not quad-tree splitting **then**7:    calculat RD cost of primary transform RD_pri8:    **if** $RD_{p}\leq \sum_{i=1}^{N-1}RD_{i}+RD\_pri$ **then**9:        isSkipMTS=true10:    **end if**11:**else**12:    calculate RD costs of primary transform RD_pri and MTS RD_mts13:    **if** $RD_{p}\leq \sum_{i=1}^{N-1}RD_{i}+min\left(RD\_pri,RD\_mts\right)$ **then**14:        isSkipMTS=true15:    **end if**16:**end if**17:return isSkipMTS

### 3.2. MTS Early Termination Method

**Figure 2:**
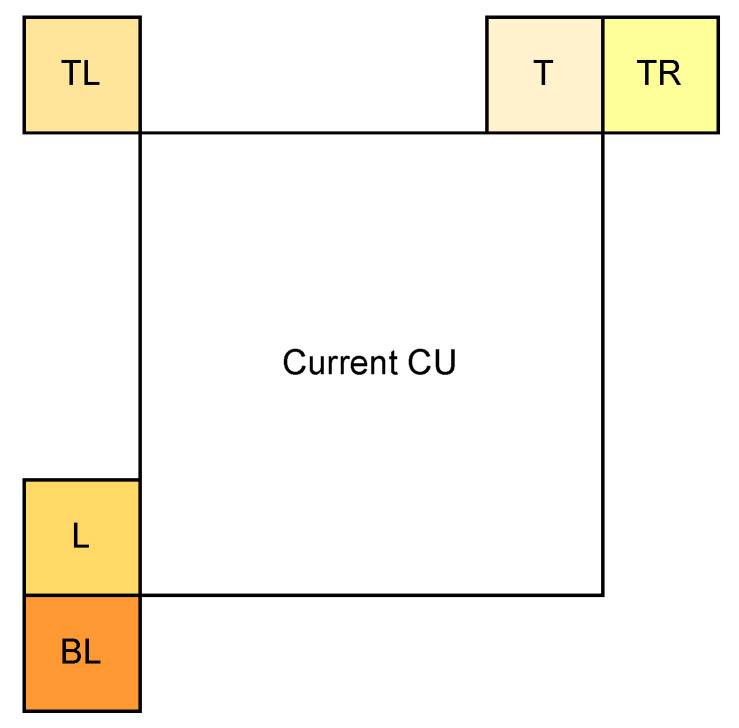
Illustration of location between the current CU and neighbouring CUs.

In the process of determining the optimal intra-mode, some of the 67 intra-modes are selected using the Rough Mode Decision (RMD) for subsequent RD checking. For these modes, the  DCT-II and MTS candidates are checked in turn, except for some candidates that the fast algorithm could skip in the Video Test Model (VTM). To accelerate the MTS selection process, we propose adjusting the above procedure of RD checking. Usually, the currently encoded CU is related to the neighboring CUs in some way, and a more reasonable algorithm can be proposed by taking full advantage of these characteristics. In order to analyse the correlation between the current CU and neighbouring CUs in terms of optimal transform, we counted the probability of using the same optimal transform for the current CU and neighbouring CUs for a large number of videos at different resolutions. The position of the current CU in relation to the neighbouring CUs is displayed in Figure 2. The L, TL, BL, T and TR represent the CUs adjacent to the left, top left, bottom left, top and top right of the current block, respectively. The MTS candidate index of these neighbouring CUs can easily be obtained if their MTS CU-Level flag is 1.

Table 3 shows the statistical probability of using the same transform between the current CU and its neighbouring CUs for all frames in a portion of the test video sequences. The QP is set at 22, 27, 32, and 37. $P_{DCT-II}$ indicates the probability that the optimal transform of the current CU is DCT-II when the optimal transform mode of all neighbouring CUs is DCT-II; $P_{MTS}$ represents the probability that the optimal transform of the current CU is MTS when the optimal transform modes of neighbouring CUs contain MTS. We can observe a strong correlation in the optimal transform between the current CU and its neighbouring CUs. In most cases, the optimal transform is included in the transforms of neighboring CUs.

Based on the above statistics and analysis of the correlation of the optimal transform between the current CU and neighboring CUs, we propose a new order of MTS candidate selection. Specifically, the proposed MTS early-termination method can be divided into two cases for the CU transforms ordering:

(1) If MTS is not included in the transform sets of the neighbouring CUs, only DCT-II is performed on the selected intra-mode.
(2) If MTS is used in the neighbouring CUs, DCT-II is first executed for the current CU, then the transform set is ranked from high to low according to the frequency of each transform in the MTS candidates used in the neighbouring CUs (the set of unused transforms is ranked after the set of used transforms in the original order). When the RD cost of the current transform is larger than the previous one, the subsequent MTS process is terminated early. After determining the best transform, the optimal prediction mode is obtained by RD checking of the prediction modes list. The overall MTS early termination method is specified in Algorithm 2.

**Algorithm 2 **The proposed MTS early termination method**Input: **the prediction modes list *L* **Output: **the minimum RD cost of second pass
$RDCost_{min}$ and the best results
1:initialize the minimum RD cost of MTS $RDCost_{minmts}$2:reorder MTS candidates according to the frequency of transform used in neighboring CUs3:calculate the RD cost of the current CU $RDCost_{min}$ using DCT-II4:**for** each transform $trans_{j}$ in MTS candidates **do**5:    **if** all neighboring CUs choose DCT-II **then**6:        break;7:    **end if**8:    **for** each predicition mode $mode_{i}$ in list *L* **do**9:        derive the RD cost $RD(mode_{i},trans_{j})$;10:        **if** $RD(mode_{i},trans_{j})$ <$RDCost_{minmts}$ **then**11:           update $RDCost_{minmts}$ by $RD(mode_{i},trans_{j})$;12:           load the prediction results of $mode_{i}$;13:        **else**14:           contine;15:        **end if**16:    **end for**17:    **if** $RDCost_{min}$>$RDCost_{minmts}$ **then**18:        update $RDCost_{min}$ by $RDCost_{minmts}$;19:    **else**20:        break;21:    **end if**22:**end for**23:return $RDCost_{min}$


## 4. Results

### 4.1. Experimental Settings

To verify the improvement of the proposed low-complexity multiple transform selection combined with a multi-type tree partition algorithm, we implemented our method in the VVC reference software VTM-3.0 and conducted experiments under JVET Common Test Conditions (CTC) [44]. The simulation used All-Intra (AI) main configuration, and the QP was set to 22, 27, 32, 37. The details of the simulation environments are shown in Table 4. In addition, the details of the open-source test video sequences are shown in Table 5. We validated the effectiveness of the proposed algorithm through extensive experiments, including comparisons with the default VTM-3.0 and state-of-the-art fast methods. The experiments were performed on an Intel core i5-3470 CPU. The coding performance of the proposed low-complexity multiple transform selection combined with multi-type tree partition algorithm was measured by the Bjøntegaard Delta Bitrate (BDBR) [45], in which negative values indicate a performance improvement. The Bjøntegaard Delta Peak Signal-to-Noise Rate (BD-PSNR) [45] is another objective index used to evaluate coding performance, in which positive values indicate performance improvements. Furthermore, the average savings of the coding time $Sav_{T}$ compared to the original VVC were calculated by:

(7) $$Sav_{T}=\frac{T_{d}-T_{p}}{T_{d}}\times 100\%,$$

where $T_{d}$ reperesents the total coding time of the VVC encoder. $T_{p}$ is the total coding time of the proposed algorithm.

### 4.2. Experimental Results and Analyses

In this subsection, the objective results of the proposed algorithm are compared with the original VVC. Table 6 shows the coding time savings by the proposed algorithm compared with the original VVC. The BDBR, which measures the coding performance of the model, is also included. Table 6 illustrates the great gain in coding speed obtained by the proposed algorithm. The results distinctly show that the proposed algorithm achieves 26.40% coding time savings on average. Compared with the original VVC, the proposed algorithm has a smaller computational complexity, making it suitable for real-time sensor applications. The BDBR only increases by 0.13%, which means that the proposed algorithm hardly degrades the coding performance of the VVC encoder. This is because the proposed algorithm fully uses the correlation between sub-CUs and the relationship between the RD cost of primary transform and MTS, so that the RD cost of the last child can be estimated more accurately and reasonably to reduce the computational complexity. Moreover, the proposed algorithm adaptively ranks the MTS candidates based on the neighboring CU information to terminate the MTS process early while ensuring that the optimal transform is not skipped in most cases.

Moreover, we also compared the proposed algorithm with the state-of-the-art fast methods. As the results shown in Table 7, the proposed low-complexity multiple transform selection combined with the multi-type tree partition algorithm saves more coding time. The experimental results demonstrate that the proposed algorithm achieves greater reductions in computational complexity without significantly increasing the BDBR. Furthermore, compared with Fu et al. [43], the proposed method achieves a minor BDBR increase, which means that the coding performance of the proposed algorithm is more reduced. Compared with previous studies [43,46], the proposed algorithm can successfully find a trade-off between encoding complexity and encoding efficiency. As we understand it, one reason for this is that the proposed algorithm reduces computational complexity by estimating the RD cost of the last child CU based on the RD cost of the previous one and their information entropy ratio. Moreover, the proposed algorithm reorders the transform candidates according to their frequency of use in the neighbouring CU. The MTS can be terminated earlier to further reduce the computational complexity by comparing the RD cost of the current transform with the minimum RD cost. Another reason for this is that the CU contents with quad-tree splitting are more diverse and complex, and the proposed algorithm does not modify the coding process of the original VVC in this case, to ensure a better coding performance.

**Figure 3:**
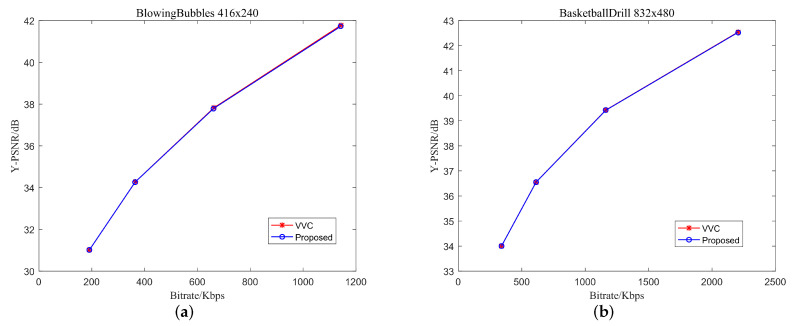
The R-D curves of sequences “BlowingBubbles” (Class D) and “BasketballDrill” (Class C) under AI configuration. (**a**) BlowingBubbles; (**b**) BasketballDrill.

To more intuitively show the effect of the proposed algorithm on the performance of VVC coding, the R-D curves of the test sequences encoded by the proposed algorithm and the original VVC are given in Figure 3. We can observe that the proposed algorithm achieves almost the same coding performance as the original VVC.

**Figure 4:**
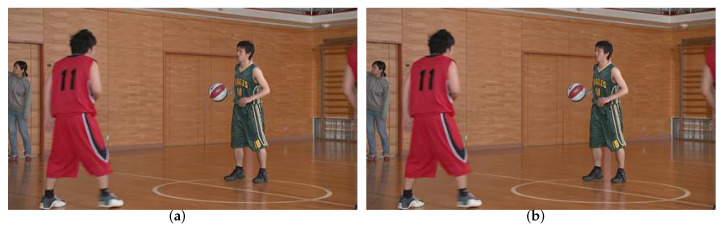
Subjective quality comparison of the first decoding frame of “BasketballPass” from Class D. (**a**) original VVC; (**b**) proposed algorithm.

Moreover, Figure 4 compares the subjective quality of the “BasketballPass” encoded by the original VVC and the algorithm proposed in this paper when QP is 22 under AI configuration. As shown in Figure 4, the differences in subjective quality between the original VVC and the proposed algorithm are also barely visible to the eyes, which indicates that the subjective quality loss caused by the algorithm proposed in this paper is negligible.

Overall, the above results demonstrate that the proposed low-complexity multiple transform selection combined with the multi-type tree partition algorithm can achieve significant coding time savings without significantly degrading the coding quality.

## 5. Conclusions

The newly added coding tool with complex calculation is the bottleneck in the implementation of VVC for real-time transmission in sensor networks. In order to save coding time and make VVC more suitable for real-time applications, we propose a low-complexity multi-transform selection combined with a multi-type tree segmentation algorithm for VVC in this paper. Based on the similarity between sub-CUs under binary splitting and the correlation between the RD cost of primary transform and MTS, a method of estimating the RD cost of the last child CU is proposed. Furthermore, when the sum of children CUs’ RD costs in the split pattern is greater than or equal to the RD cost of the parent CU, the RD checking of MTS is skipped. To further accelerate the coding process, an MTS early termination method is proposed. The RD calculation for some MTS candidates is terminated in advance by making full use of the coding information of neighbouring CUs. Experimental results demonstrate that, compared with the original VVC, the proposed algorithm achieves time savings of 26.4% on average, while maintaining a similar coding performance. In future work, we will focus on fast prediction modes and CU partitioning methods in VVC and combine them with the proposed low-complexity MTS method to achieve more coding time savings.

## Figures and Tables

**Table 1:** The mapping relationship between MTS indexes and transforms.

MTS Candidate Indexes	Horizontal	Vertical
0	DST-VII	DST-VII
1	DST-VII	DCT-VIII
2	DCT-VIII	DST-VII
3	DCT-VIII	DCT-VIII

**Table 2:** The probability of using the same optimal transform in two adjacent sub-CUs under binary splitting.

Video Sequences	Probability
BasketballPass	94.7%
RaceHorsesC	93.6%
Johnny	96.7%
BasketballDrive	95.5%
Average	95.1%

**Table 3:** The probability of being able to use the same optimal transform between the current CU and its neighboring CUs.

Video Sequences	$P_{\mathrm{DCT}-II}$	$P_{\mathrm{MTS}}$
BasketballPass	88.5%	78.6%
RaceHorsesC	80.6%	82.7%
Johnny	82.3%	79.3%
BasketballDrive	85.6%	80.5%
Average	84.3%	80.3%

**Table 4:** The environments and conditions of simulation.

Items	Descriptions
Software	VTM-3.0
Configuration File	encoder intra vtm.cfg
Video Sequence Size	416 × 240, 832 × 480,
	1280 × 720, 1920 × 1080
Number of Encoded Frames	30
Quantization Parameter (QP)	22, 27, 32 and 37
Sampling of Luminance to Chrominance	4:2:0

**Table 5:** Detailed characteristics of the experimental video sequences.

Class	Sequences	Size	Bit-Depth	Frame Rate
	BasketballDrive	1920 × 1280	8	50
B	BQTerrace	1920 × 1280	8	60
	Cactus	1920 × 1280	8	50
	BasketballDrill	832 × 480	8	50
C	BQMall	832 × 480	8	60
	PartyScene	832 × 480	8	50
	BasketballPass	416 × 240	8	50
D	BlowingBubbles	416 × 240	8	50
	RaceHorses	416 × 240	8	30
	FourPeople	1280 × 720	8	60
E	Johhny	1280 × 720	8	60
	KristenAndSara	1280 × 720	8	60
	Slideshow	1280 × 720	8	20
F	SlideEditing	1280 × 720	8	30
	BasketballDrillText	832 × 480	8	50

**Table 6:** The proposed algorithm compared to the original VVC experimental results.

Class	Sequences	BDBR/%	BD-PSNR/db	${\mathrm{Sav}}_{T}$/%
	BasketballDrive	0.08	−0.007	29.16
B	BQTerrace	0.10	−0.004	28.02
	Cactus	0.15	−0.008	26.06
	BasketballDrill	0.12	−0.007	27.42
C	BQMall	0.09	−0.003	24.53
	PartyScene	0.10	−0.009	29.30
	BasketballPass	0.15	−0.007	24.18
D	BlowingBubbles	0.12	−0.008	27.09
	RaceHorses	0.14	−0.007	25.13
	FourPeople	0.16	−0.006	26.89
E	Johhny	0.14	−0.007	25.47
	KristenAndSara	0.11	−0.005	29.40
	Slideshow	0.18	−0.009	24.64
F	SlideEditing	0.14	−0.008	22.35
	BasketballDrillText	0.13	−0.008	26.42
Average	-	0.13	−0.007	26.40

**Table 7:** The proposed algorithm compared to the state-of-the-art experimental results.

Sequences	Fu et al. [43]		Zhang et al. [46]		Proposed	
	**BDBR(%)**	${\mathrm{Sav}}_{T}$ **(%)**	**BDBR(%)**	${\mathrm{Sav}}_{T}$ **(%)**	**BDBR(%)**	${\mathrm{Sav}}_{T}$ **(%)**
Cactus	0.18	23	−0.01	10	0.15	26.06
BQTerrace	0.12	25		8	0.10	28.02
BasketballDrive	0.09	23		8	0.08	29.16
BQMall	0.11	24	0.02	3	0.09	24.53
PartyScene	0.16	25		5	0.10	29.30
BasketballDrill	0.14	21		9	0.12	27.42
BasketballPass	0.19	23	0.06	7	0.15	24.18
BlowingBubbles	0.17	24		6	0.12	27.09
RaceHorses	0.16	23		1	0.14	25.13
FourPeople	0.22	23	0.03	7	0.16	26.89
KristenAndSara	0.19	23		10	0.11	29.40
Johnny	0.2	22		9	0.14	25.47
Average	0.16	23.30	0.03	6.92	0.12	26.89

## Data Availability

Not applicable.
